# 

**Published:** 1834-04-01

**Authors:** 


### CONTENTS

OF THE

### MEDICAL QUARTERLY REVIEW,

No. III. APRIL 1, 1834.

### REVIEWS.

I.

Lectures on the Morbid Anatomy, Nature, and Treatment of Acute and Chronic Diseases, delivered in the Theatre of Anatomy, Webb street, by the late JOHN ARMSTRONG, M.D. Edited by JOSEPH RIX . 1

II.

Erfahrungen über die Erkenntniss, und Heilung der langwierigen Schwerhörigkeit. Von Dr. W. KRAMER . . . 17

III.

On the Reflex Function of the Medulla Oblongata and Medulla Spinalis. By MARSHALL HALL, M.D., F.R.S. L. & E., &c. . . 25

IV.

Elements of Materia Medica and Therapeutics; including the recent Discoveries and Analyses of Medicines. Vol. II. By ANTHONY TODD THOMSON, M.D. . . . . 39

V.

Alphabet of Medical Botany, for the Use of Beginners. By J. RENNIE, M.A. &c. . . . . . 50

Alphabet of Botany, for the Use of Beginners. By J. RENNIE, M.A. &c. ib.

VI.

An Investigation into the remarkable Medicinal Effects resulting from the External Application of Veratria. By ALEXANDER TURNBULL, M.D. 55

VII.

The Medical Works of PAULUS ÆGINETA, the Greek Physician, translated into English; with a copious Commentary, containing a comprehensive View of the Knowledge possessed by the Greeks, Romans, and Arabians, on all Subjects connected with Medicine and Surgery. By FRANCIS ADAMS, Esq. . . . . 60

VIII.

The Cyclopædia of Practical Medicine. Edited by JOHN FORBES, M.D., ALEXANDER TWEEDIE, M.D., and JOHN CONOLLY, M.D.

Dr. CARSWELL on the Softening of Organs . . 78

Dr. PRICHARD on Somnambulism and Animal Magnetism . ib.

Dr. BISSET HAWKINS on Medical Statistics . . 81

Dr. WILLIAMS on the Stethoscope . . . ib.

Dr. A. T. THOMSON on Stimulants . . . 82

*Reviews continued.*

IX.

Die Geburtshülfliche Exploration. Von Dr. Anton Friedrich Hohl 83

X.

A Treatise on Diseases and Injuries of the Nerves. By Joseph Swan, Esq. A new Edition, very considerably enlarged . . 90

XI.

The Croonian Lectures, delivered at the Royal College of Physicians, in 1833, on Cholera. By George Leith Roupell, M.D. . 97

XII.

A Treatise on the Diseases of Newly-born Infants, and of their Diseases during Lactation. By C. M. Billard, D.M. Second Edition, enlarged by a Medico-legal Memoir on the Vitality of the Fœtus, with Notes, and a Notice of the Life and Works of the Author, by M. Ollivier (d'Angers) . . . . 111

XIII.

On the Influence of Minute Doses of Mercury, combined with the appropriate Treatment of various Diseases, in restoring the Functions of Health; and the Principles on which it depends. By A. P. W. Philip, M.D., F.R.S. L. & E., &c. . . . . 121

XIV.

Cases illustrating and confirming the Remedial Power of Inhalation of Iodine and Conium in Tubercular Phthisis, and various disordered States of the Lungs and Air-Passages. By Sir C. Scudamore, M.D. 127

XV.

Illustrations of the Elementary Forms of Disease. By Robt. Carswell, M.D. Fasciculi I.—IV. . . . . 131

XVI.

A Demonstration of the Nerves of the Human Body; consisting of four Parts. By Joseph Swan. Part IV. . . . 134

XVII.

Report of the Experiments on Animal Magnetism, &c. Translated, with an Historical and Explanatory Introduction, and an Appendix, by J. C. Colquhoun, Esq. . . . . 135

XVIII.

Observations on the Ulcerative Process, and its Treatment, particularly when affecting the Leg. By Wm. Eccles, Surgeon . 137

XIX.

An Examination into the Causes of the declining Reputation of the Medical Faculty of the University of Edinburgh, &c. . . 139

XX.

Medical Bibliography. A. & B. By James Atkinson, Surgeon 141

XXI.

Observations on the Preservation of Sight, and on the Use, Abuse, and Choice of Spectacles, &c. By John Harrison Curtis, Esq. 144

*Reviews continued.*

XXII.

The Anatomy and Surgery of Inguinal and Femoral Hernia. By E. W. TUSON, F.L.S. . . . . . 145

XXIII.

A Series of Anatomical Plates in Lithography, with References and Physiological Comments, illustrating the Structure of the different Parts of the Human Body. Edited by JONES QUAIN, M.D. . 146

XXIV.

Some Observations on the Utility of Fumigating and other Baths, &c. By JONATHAN GREEN, M.R.C.S. . . . ib.

XXV.

On Dentition, and some coincident Disorders. By J. ASHBURNER, M.D. 147

XXVI.

An Introduction to the Study of Human Anatomy. By JAMES PAXTON, Esq. 148

XXVII.

The Journal of Botany. By WM. JACKSON HOOKER, Esq. . 149

XXVIII.

An Essay on the Physiology of the Iris. By JOHN WALKER, Esq. 150

XXIX.

The Parents' Dental Guide, &c. By WM. IMRIE, Esq. . ib.

XXX.

An Introduction to the Study and Practice of Medicine. By JOHN DOWSON, Esq. . . . . . 151

### Original Communications.

I.

On the Classification, Administration, Modus Operandi, and Combination of Medicines. By J. STEVENSON BUSHNAN, F.L.S. . 152

II.

Account of a Case of Pulmonary Consumption, in which nearly the whole of the Right Lung was converted into an immense Vomica, attended with universal Adhesion, and partial Absorption of the Pleural Sac. Communicated to the Harveian Society, by WILLIAM STROUD, M.D. 170

III.

Cases extracted from the Obstetric Note-book of the Welbeck-street Dispensary, by permission of HENRY DAVIES, M.D. . 179

IV.

On the Use of Colchicum in Erysipelas. By JOHN BULLOCK, Esq. 183

### COLLECTANEA.

### PATHOLOGY AND PRACTICE.

On the Application of the Tourniquet in Paralysis . . 188
Case in which the Os Uteri was nearly Obliterated . . 189
Case of Hypochondriasis . . . . . ib.
Hypochondriasis in Medical Pupils . . . 190
Paralysis of one Side of the Face . . . ib.
Empyema cured by an Operation. By J. PANCOAST, M.D. . 191
Chorea Sancti Viti . . . . . 196
Lithotomy in Italy . . . . . ib.
Cases of Diseased Nerves . . . . 200
Cases of Dilatation of the Stomach . . . 201
Case of Constipation, successfully treated by the Introduction of Air into the Bowels. By GEORGE J. JANEWAY, M.D. . . 203
Treatment of Dropsy . . . . . 204
On the Crowing Inspiration of Children . . . 205
Therapeutic Effects of Codeine . . . 206

### MISCELLANEOUS.

Letter from Mr. MACILWAIN . . . . 207
Regulation of Madhouses . . . . 208
Great Oaks . . . . . 211
On the Apparent Direction of Eyes in a Portrait . . 212
The Weeping Willow . . . . . 213
On the Structure of the Covering of the Cornea . . 215
Test for Hydrocyanic or Prussic Acid, and Method of Appreciating the Quantity . . . . . 216
Spark produced during the Freezing of Water by Æther . . 217
Horse Shoes . . . . . 218
A Surgical Anecdote . . . . ib.
On Sounds Inaudible by certain Ears . . . 220
The Taxodium . . . . . 221
On the Organs of the Voice in Birds . . . ib.
The Hippomaneæ . . . . 226
Case of Ulceration of the Eyelid . . . 227
Homœopathy . . . . . 228
Piscidia Erythrina, or the Fish-wood . . . 230
Reduction of Strangulated Hernia . . . . 231
Fœtus of the Whale . . . . . ib.

### Medical Politics and Intelligence.

Medical Reform . . . . . 232
College of Physicians . . . . 234
Oxford Medical Degrees . . . . ib.
Statutes of the University of Edinburgh, relative to the Degree of M.D. 1833 2[illegible]8
List of Questions to be answered by the Governors of Hospitals, &c. 240
British Association for the Advancement of Science . ib.
Meteorological Register. Notices, &c. . . . ib.

---

**Advertising Sheet—(see End of the Number.)**

